# Growth and differentiation of primary and passaged equine bronchial epithelial cells under conventional and air-liquid-interface culture conditions

**DOI:** 10.1186/1746-6148-7-26

**Published:** 2011-06-07

**Authors:** Getu Abraham, Claudia Zizzadoro, Johannes Kacza, Christin Ellenberger, Vanessa Abs, Jana Franke, Heinz-Adolf Schoon, Johannes Seeger, Yohannes Tesfaigzi, Fritz R Ungemach

**Affiliations:** 1 Institute of Pharmacology, Pharmacy and Toxicology, Faculty of Veterinary Medicine, University of Leipzig, An den Tierkliniken 15, 04103 Leipzig, Germany; 2 Department of Anatomy, Histology and Embryology, Faculty of Veterinary Medicine, University of Leipzig, An den Tierkliniken 43, 04103 Leipzig, Germany; 3 Institute of Pathology, Faculty of Veterinary Medicine, University of Leipzig, An den Tierkliniken 33, 04103 Leipzig, Germany; 4 Lovelace Respiratory Research Institute, 2425 Ridgecrest Dr., SE, Albuquerque, NM 87108, USA

## Abstract

**Background:**

Horses develop recurrent airway obstruction (RAO) that resembles human bronchial asthma. Differentiated primary equine bronchial epithelial cells (EBEC) in culture that closely mimic the airway cells *in vivo *would be useful to investigate the contribution of bronchial epithelium in inflammation of airway diseases. However, because isolation and characterization of EBEC cultures has been limited, we modified and optimized techniques of generating and culturing EBECs from healthy horses to mimic *in vivo *conditions.

**Results:**

Large numbers of EBEC were obtained by trypsin digestion and successfully grown for up to 2 passages with or without serum. However, serum or ultroser G proved to be essential for EBEC differentiation on membrane inserts at ALI. A pseudo-stratified muco-ciliary epithelium with basal cells was observed at differentiation. Further, transepithelial resistance (TEER) was more consistent and higher in P_1 _cultures compared to P_0 _cultures while ciliation was delayed in P_1 _cultures.

**Conclusions:**

This study provides an efficient method for obtaining a high-yield of EBECs and for generating highly differentiated cultures. These EBEC cultures can be used to study the formation of tight junction or to identify epithelial-derived inflammatory factors that contribute to lung diseases such as asthma.

## Background

The bronchial epithelium has been shown not only to serve as physicochemical barrier to inhaled chemical or physical substances and infectious agents but also to be involved in the pathogenesis of respiratory diseases in humans and animals. For example, airway epithelial cells play a pivotal role in the patho-mechanisms of human asthma, chronic obstructive pulmonary diseases (COPD) [1] and inherited disease such as cystic fibrosis [2,3]. They initiate and augment the innate immunity by forming and releasing pro- or anti-inflammatory mediators that can alter cell differentiation, chemotaxis, and activation of immune cells [4]. Thus, in standard animal models of asthma such as in mice, rats, guinea pigs, rabbits and dogs airway epithelial cells have been considered as pathological and therapeutic targets of airway diseases.

Among large animal models, horses show naturally occurring airway disease i.e. recurrent airway obstruction (RAO), also known as "heaves", displaying many of the hallmark features of allergic airway disease in humans [5-7]. As large animal models, horses would offer a wider scope for repeated sampling and measures of airway function and unlike human studies allow for greater control and study in the onset, progression and resolution of experimental equine, and presumably human, airway diseases [8,9]. Therefore, establishing and characterizing an equine bronchial epithelial cell (EBEC) culture system can provide the opportunity to investigate more closely the possible contribution of epithelial cell biology to RAO in horses.

Despite several attempts of isolating and culturing horse airway epithelial cells [10,11], their role in the pathogenesis of RAO has not been investigated. This was presumably hampered because the culture conditions did not fully resemble the *in vivo *conditions in terms of differentiation and polarization; properties that are observed only when these cells are cultured under air-liquid-interface (ALI) conditions [12]. Current reports on culturing equine bronchial epithelial cells merely described how to get confluent cultures on solid supports in serum-free medium, but the degree of cell differentiation which limits their use for *in vitro *studies was not evaluated [10]. The only study attempting to establish ALI-cultures in serum-free medium [11] did not characterize the state of EBEC differentiation and polarization using measurements for trans-epithelial resistance (TEER), tight junction expression, and cell-type specific expression of cytokeratins, all markers of differentiation resembling *in vivo *airway epithelial conditions. It is known that tight junctions are characteristic features of differentiated airway epithelia and determine epithelial barrier as well as cell growth and differentiation.

Extensive effort has been made to establish such methods of isolation and culture of human and other laboratory animal airway epithelial cells that reliably result in cellular differentiation and polarization. Culturing primary and passaged airway epithelial cells on semi-permeable membrane supports under ALI conditions has been proven to facilitate the development of muco-ciliary and pseudo-stratified morphology [12-15]. Variability in airway epithelial cell differentiation in ALI cultures depends on whether freshly isolated and pre-cultured (on solid supports) cells were seeded. Other factors include the use of serum-free, hormone and growth factors supplemented airway epithelial cell growth medium (AECGM) or DMEM/Ham's F-12 medium supplemented with serum-like substituent such as Ultroser G [16-20]. In some studies, gene expression and basic cellular function of such ALI cultures were shown to resemble those found *in vivo *in intact bronchial epithelium [1,17].

The objective of the present study was to establish and extensively optimize EBEC isolation and culture conditions to generate cell differentiation with important prerequisites to resemble the airway *in vivo *epithelium. Primary (P_0_) and passaged (P_1_) EBECs from the same horse were grown and differentiated on solid supports or membrane inserts under ALI condition in serum-supplemented or -deficient AECGM. The level of differentiation and polarization was characterized by light and phase-contrast microscopy, standard histological staining, immunocytochemistry, scanning electron microscopy (SEM), and by measuring transepithelial electrical resistance (TEER).

## Results

### Growth and Differentiation of EBECs under conventional culture conditions

**Figure 1:**
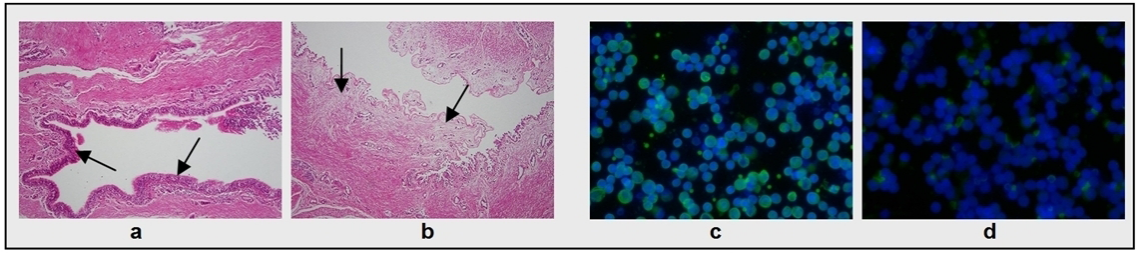
**Effectiveness of EBEC isolation procedure and purity of fresh isolates**. [a-b] Representative H&E staining of equine bronchial tissue before (a) and after (b) 2 h exposure to 0.25% trypsin-EDTA (objective magnification: ×10). Arrows indicate the intact epithelium before digestion and the complete removal of epithelium from the underlying connective tissue after trypsin digestion. [c-d] Representative immunocytochemical staining of cytocentrifuged fresh EBEC for the epithelial cell markers cytokeratins 5/6/18 (c) and the mesenchymal cell marker vimentin (d) (objective magnification: ×40). Green fluorescence (FITC) stain indicates positive signal. Nuclei are blue stained with DAPI.

Successful dissociation of epithelial cells was confirmed by H&E staining after short-term (2 h) incubation of native bronchial tissues with 0.25% trypsin-EDTA solution (Figure 1a, b). A mean yield of 15.25 ± 1.90 × 10^6 ^viable cells was obtained from 500 mg minced tissue, with viability of > 95% as assessed by trypan blue exclusion test (n = 12). Freshly dissociated cells often consisted of single or clumped and rotating ciliated cells. 92.28 ± 0.88% of these cells were CK5/6/18-positive indicating epithelial origin while only 9.43 ± 1.13% (n = 12) VIM-positive suggesting non-epithelial cell origin (Figure 1c, d). 100 cells were counted in at least three randomly selected fields.

**Figure 2:**
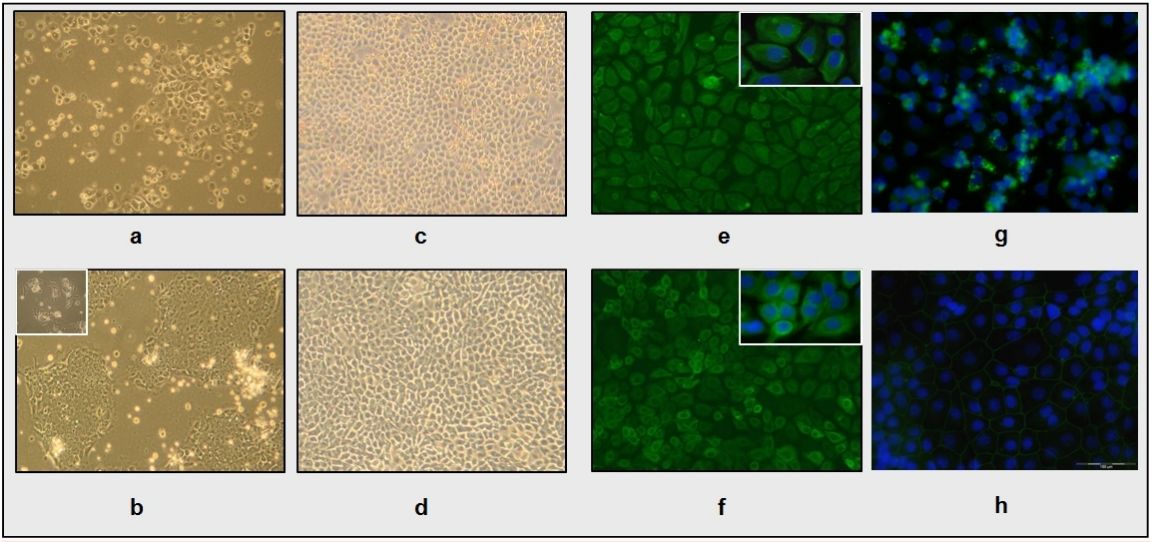
**Morphological and immunocytochemical appearance of EBEC cultures on solid supports in serum-free and serum-containing medium**. [a-b/c-d] Phase contrast microscopy of EBEC cultures 2 days after plating (objective magnification: ×10) and at confluence (objective magnification: ×4) in serum-free (a-c) and serum-containing (b-d) medium. Inset in picture b shows single cells grown in the presence of serum forming fine interconnecting cytoplasmic bridges (objective magnification: ×40). [e-f] Representative immunocytochemical staining of confluent EBEC cultures grown on glass cover slips for the epithelial cell markers cytokeratins 5/6/18, showing localization of the green positive signal in the cytoskeleton of cells grown in both serum-free (e) and serum-containing (f) medium (objective magnification: ×20). In the insets, nuclei are blue stained with DAPI (original objective magnification: × 40). [g-h] Representative immunocytochemical staining of confluent EBEC cultures grown on glass cover slips for the tight junction protein ZO-1, showing spot-like green fluorescent signal in cytoplasm of cells cultured in serum-free medium (g) and circumferential localization of the green positive signal in cells cultured in serum-containing medium (h) (objective magnification: ×40). Nuclei are blue stained with DAPI.

When cells were seeded at a density of 5 × 10^5^/cm^2 ^on collagen-coated flasks, 77.16 ± 1.76% (n = 5) and 99.17 ± 0.73% (n = 3) of EBECs could attach after 24 h in serum-containing and serum-free AECGM, respectively. We have tested FCS at concentrations of 5 and 2.5% but were not able to reproducibly generate healthy cultures under LLI or ALI conditions; therefore, we decided to focus on using 10% FCS that showed optimal growth. This suggests that 10% serum leads to a reduction in cell attachment efficiency. Serum also affected cell morphology and growth pattern (Figure 2). EBECs cultured in serum-free medium showed distinct polygonal shape with clear margins and typically formed lines and chains while spreading over the growth surface (Figure 2a). Cells grown in serum-containing AECGM had more compact growth pattern and a marked tendency for cell-cell-contacts, as indicated by the presence of fine cytoplasmic bridges connecting single cells as well as the boundaries of cell colonies (Figure 2b). Moreover, these colonies formed large coherent sheets with poorly defined margins between single cells and acquired distinct and typical epithelial polygonal shape but only in confluent areas. Addition of serum initially stimulated faster proliferation compared to cultures in serum-free AECGM. However, cells cultured in the presence or absence of serum required similar median days (4.5 or 5.5 days, respectively) to reach 80-100% (n = 8) confluence.

After cultures reached complete confluence, no apparent morphological differences were observed among cells grown with or without serum. In both types of media, confluent monolayers formed typical epithelial mosaic-like or cobblestone appearance (Figure 2c, d), with 100% CK-5/6/18-positive cells (Figure 2e, f) and no detectable signal for VIM (data not shown). However, immuncytochemical staining for the tight junction protein ZO-1 showed different patterns: cells grown in serum-free AECGM showed only spot-like cytoplasmic immunopositive green signal (Figure 2g), while cells grown in serum-containing AECGM showed continuous immunopositive signal (Figure 2h).

**Figure 3:**
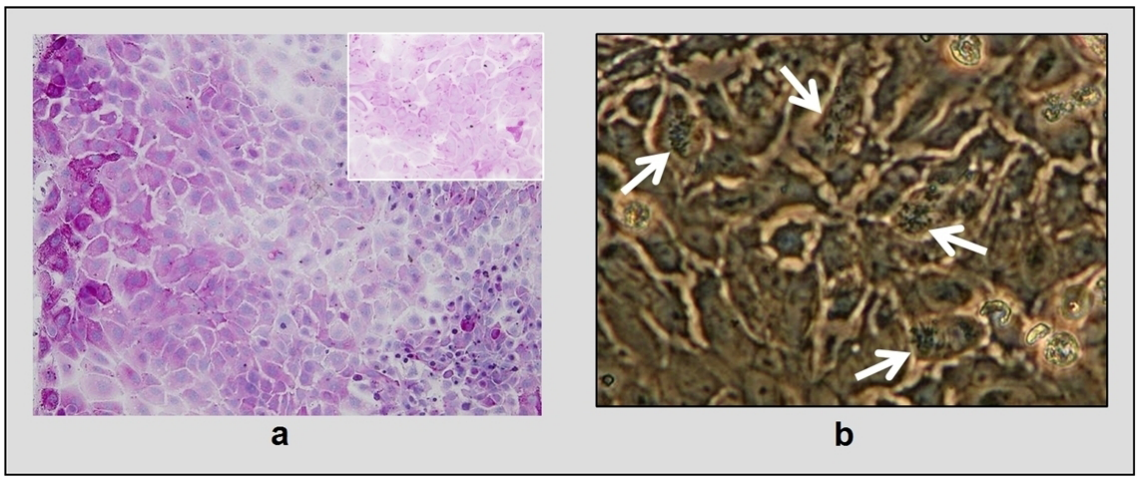
**Features of mucociliary differentiation of EBEC cultured on solid supports in serum-free or serum-containing medium**. [a] PAS-positive (magenta coloured) cells in 30 days old EBEC cultures on glass cover slips in serum-containing medium (objective magnification: ×10); inset shows less intense PAS-positive stain in EBEC grown in serum-free medium (objective magnification: ×40). [b] Ciliated cells detected by phase contrast microscopy in 30 days old EBEC cultures on tissue culture flasks and glass cover slips in serum-containing medium; arrows indicate cilia (objective magnification: ×40).

In addition to the observed formation of tight junctions, EBEC cultures in serum containing AECGM exhibited also more typical features of differentiation (Figure 3). Many PAS-positive cells (likely mucus-producing cells) (Figure 3a) as well as many ciliated cells were routinely detected (Figure 3b; video images can be supplied upon request), even 24 h after plating and during the whole culture period (~ 30 days). In contrast, in serum-free AECGM, EBECs completely lacked visible cilia and showed less intense PAS-positivity (Figure 3a - inset).

When primary (P_0_) EBECs were sub-cultured at a density of 5 × 10^5^/cm^2 ^on collagen-coated plastic supports, passage was possible for up to P_2_. Compared to P_0 _cultures, the P_1 _and P_2 _cultures usually required more than 10 d to reach 80-100% confluence in both serum-free and serum-containing media. However, subculture was more successful when cells were grown in serum-free AECGM. Markers of differentiation, i.e. PAS-positive and ciliated cells were significantly reduced in P_1 _cultures, and ciliated cells were completely absent in P_2 _cultures regardless of serum.

### Growth and Bioelectrical Properties of P_0 _and P_1 _EBEC ALI-Cultures

P_0 _and P_1 _cells obtained from 6 different horses were used for establishing cultures on collagen-coated membrane inserts under ALI conditions. Interestingly, when these cells were seeded and maintained under submerged condition (LLI) in serum-free AECGM, they failed to proliferate and effectively develop tight junctions. Moreover, TEER measurements were not different from basal resistance measured in cell-free inserts. In contrast, when serum-containing AECGM was used in liquid-liquid- and ALI conditions, both P_0 _and P_1 _cultures formed tight junctions and cells were polarized and differentiated into a pseudo-stratified and mucociliary epithelium.

**Figure 4:**
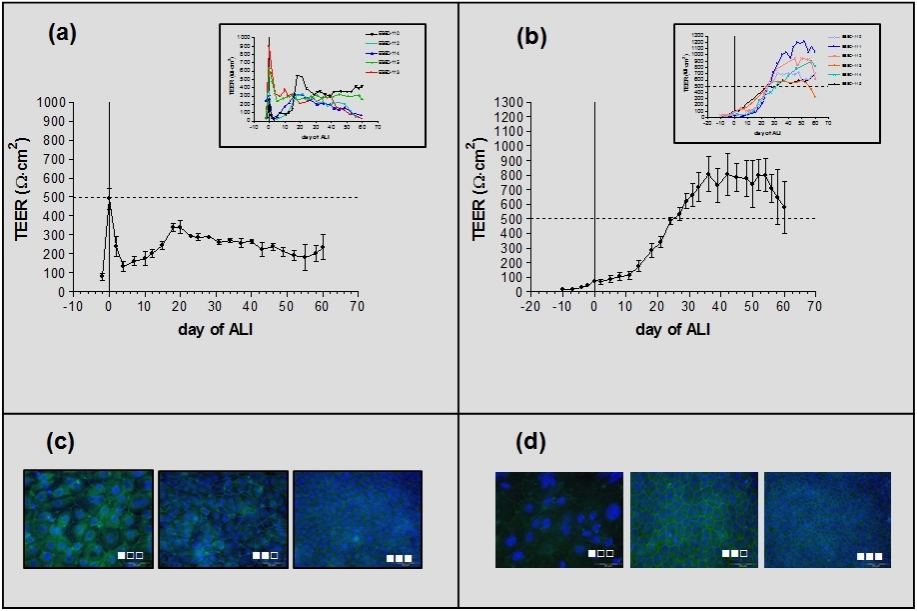
**TEER measurements and tight junction detection in P_0 _and P_1 _EBEC cultures at ALI**. [a-b] Time-course of TEER development in P_0 _(a) and P_1 _(b) insert cultures; the *abscissa *indicates culture days before (negative) and after (positive) ALI creation (day 0); TEER values (Ω·cm^2^) at each time-point are expressed as mean ± SEM of n = 5 (P_0_) or n = 6 (P_1_); insets show TEER graphs of individual subjects. [c-d] Representative images of time-dependent immunofluorescence staining of P_0 _(c) and P_1 _(d) insert cultures for the tight junction protein ZO-1 (*one black and two white squares*: ALI day 0, immediately before ALI creation; *two black and one white squares*: intermediate time-point of the ALI-culture phase leading to bioelectrical stabilization; *all black squares*: mature and electrically stable ALI-cultures) (objective magnification: ×40). Nuclei are blue stained with DAPI.

P_0 _insert cultures were successfully established from all 6 horses, although marked inter-individual variations could be observed. The data from one horse differed largely from the others and, thus, was excluded. Under liquid-liquid-interface (LLI) condition, cells at P_0 _showed a high rate of proliferation and usually formed confluent layers within 3-5 d post-seeding. TEER increased rapidly and in a time-dependent manner and reached mean values of 490.87 Ω·cm^2 ^(± 122.45; n = 5) at confluence (Figure 4a). When cultures were switched to ALI conditions (d 0), TEER decreased abruptly to 79.16 ± 6.82% (n = 5) between d 0 and 4. In three cultures, which had TEER values ranging between 247.18 and 352.91 Ω·cm^2 ^(Figure 4a - inset), the switch to ALI induced pronounced decrease in TEER (90.08 ± 2.34% at d 1-3 post-ALI). The decrease in TEER values was accompanied with partial retraction of the cell layer forming holes and at later time points with retraction of the entire cell layer at the periphery. Nevertheless, recovery of these alterations was seen starting from d 4-7 of ALI, during which cell proliferation occurred and again TEER values increased. Complete layer confluence was again achieved between d 18-21 of ALI and TEER was stable with values of about 300 Ω·cm^2^. In the remaining two cultures, TEER values of 637.00 and 902.22 Ω·cm^2 ^could be measured under LLI conditions (Figure 4a - inset) and switching to ALI resulted in a moderate but permanent TEER decrease (62.77 ± 0.82%, n = 2) without visible alteration in cell layer appearance. For these cultures, TEER values were stable at about 300 Ω·cm^2 ^between day 4 and 9 of ALI. Mean TEER values of P_0 _cultures finally stabilized at 310.53 ± 18.62 (n = 5) for up to d 30 of ALI (Figure 4a); among them two cultures remained stable even for up to 60 d (inset).

When EBECs from the same 6 horses which were primarily cultured on solid supports were seeded on collagen-coated transwell membrane inserts, all P_1 _ALI cultures were successfully established. These P_1 _cells showed more homogenous growth and bioelectrical behaviour than in P_0 _cultures. Under LLI conditions, P_1 _cells showed a slower rate of proliferation and development of bioelectrical resistance than P_0 _cells. Confluence was usually not achieved even after 12 d of culture and this was the time required for TEER to reach a threshold value of 50-100 Ω·cm^2 ^(mean TEER at ALI d 0: 75.06 ± 8.65 Ω·cm^2^; n = 6) (Figure 4b). In contrast to P_0 _cultures, switching P_1 _cultures to ALI condition did neither alter cell layer morphology nor substantially affect TEER values. Usually, starting at d 6-11 of ALI, intense cell proliferation coincided with a time-dependent increase in TEER values. TEER values of about 400-500 Ω·cm^2 ^were usually observed between day 21 and 28 of ALI and TEER increased gradually, reaching maximum values of about 789.76 ± 90.46 Ω·cm^2 ^(range: 546.70 - 1114.03 Ω·cm^2^; n = 6) by d 35. These TEER values remained stable for further 20 - 25 d (i.e. 55 - 60 d of ALI) (Figure 4b - inset), with no apparent morphological alteration of the cell layer.

### Tight junction protein (ZO-1) expression in EBEC P_0 _and P_1 _ALI cultures

Because high TEER values implied the formation of functional tight junctions, we analyzed P_0 _and P_1 _EBEC insert cultures for the expression of the tight junction protein ZO-1 (TJP1) by immunocytochemistry. In both P_0 _(Figure 4c) and P_1 _(Figure 4d) cultures we could observe continuous positive signals, lining cell boundaries, confirming the expression as well as the intercellular localization of tight-junctions.

Depending on culture conditions and culture age, changes were seen in ZO-1 expression. In LLI cultures, the continuous green fluorescent rings of ZO-1 looked large and corresponded to the numbers of green rings and DAPI-stained nuclei. In mature ALI-cultures, usually, more than one nucleus could be observed within a single fluorescent ring. Moreover, nuclear and ZO-1 stains could not be captured in the same plain of focus. The ZO-1 positive cells per field progressively increased in number but decreased in size with the age of ALI (Figures 4c, d).

### Microscopic features of P_0 _and P_1 _EBEC ALI cultures

**Figure 5:**
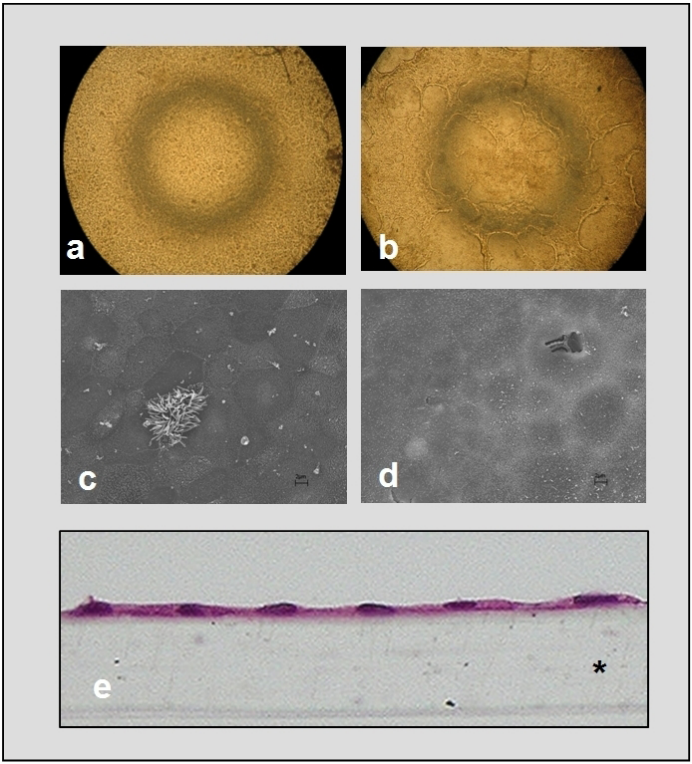
**Morphological appearance of P_0 _and P_1 _EBEC liquid-liquid interface cultures before ALI creation**. [a-b] Light microscopy of epithelial cell layers generated by freshly isolated cells (P_0 _insert cultures) (a) and by cells recovered from sub-confluent P_0 _cultures previously grown on solid supports in serum-containing medium (P_1 _insert cultures) (b) (objective magnification: ×4). [c-d] Representative scanning electron micrographs of P_0 _(c) and P_1 _(d) EBEC insert cultures: in P_0 _cultures (c) few ciliated cells, small amount of mucus and short microvilli can be observed; in P_1 _cultures (d) cells show poorly defined boundaries and many short microvilli. Scale bars = 2 μm. [e] Representative H&E staining of sections of epithelial layers grown on membrane inserts (objective magnification: ×20), showing that in both P_0 _and P_1 _cultures epithelial cells form a flat monolayer. Asterisk indicates the porous membrane support.

As assessed by light and electron microscopy, or by H&E staining and immunocytochemistry, P_0 _and P_1 _cells on membrane inserts showed similarities and differences depending on the conditions and age of the ALI culture (Figure 5). Regardless of confluence level, cells under LLI condition (Figure 5a, b), were large, uniform, with short microvilli and poorly defined borders (especially in P_1 _cultures) (Figure 5c, d) and formed a monolayered, flattened and almost undifferentiated epithelium (Figure 5e). Indeed, some ciliated cells could be observed in P_0 _cultures at this stage.

**Figure 6:**
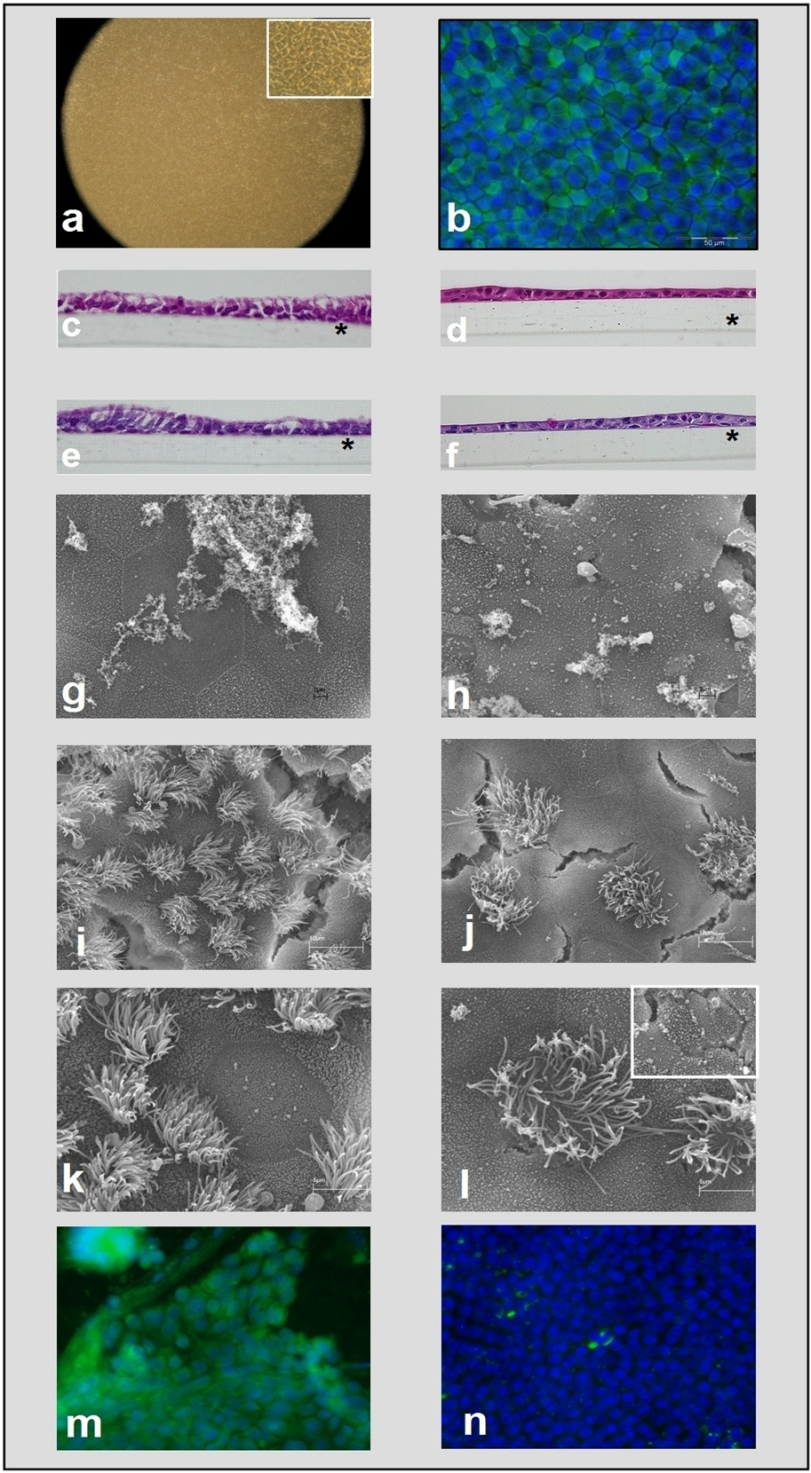
**Morphological, histological and immunocytochemical appearance of mature P_0 _and P_1 _EBEC ALI cultures**. [a] Phase contrast microscopy of mature confluent epithelial cell layers showing typical mosaic-like appearance (objective magnification: ×4), at higher magnification in the inset (×40). [b] Representative image of immunofluorescence staining of mature cultures for cytokeratins 5/6/18 (objective magnification: ×40). [c-d] Representative H&E staining of sections of epithelial layers grown on membrane inserts at P_0 _(c) and P_1 _(d) (objective magnification: ×40), indicating pseudo-stratified organization with columnar (c) or cuboidal (d) cells and flat basal cells. Asterisk indicates the porous membrane support. [e-f] PAS-positive cells in P_0 _(e) and P_1 _(f) mature EBEC insert cultures. Asterisk indicates the porous membrane support. [g-l] Representative scanning electron micrographs of mature P_0 _(g, i, k) and P_1 _(h, j, l) EBEC ALI-cultures, showing the presence of mucus-like amorphous material (g, h) (scale bars = 3 μm), cilia (i, j) (scale bars = 10 μm) and microvilli (k, l) (scale bars = 5 μm) at the apical cell surface. Inset of picture (l) shows the presence of particularly elongated microvilli-like projections in some surface areas of P_1 _cultures (scale bars = 2 μm). [m, n] Representative images of immunofluorescence staining of P_0 _and P_1 _cultures with antibodies directed against the cell type-specific cytokeratins 18 (m) and 10/13 (n) (objective magnification: ×40). Nuclei are blue stained with DAPI.

In contrast, once ALI cultures were established, cells looked better defined, smaller in size, and with typical epithelial polygonal shape (Figure 6a). Immunofluorescence staining revealed 100% positivity for CKs 5/6/18 (Figure 6b) and no signal for VIM (data not shown), confirming epithelial cell origin. Cells of mature ALI-cultures showed increased height, a columnar, monolayered pseudo-stratified epithelium (Figure 6c, d). Mature ALI-cultures were PAS-positive, suggesting the presence of mucus-producing cells (Figure 6e, f). Ciliary beating could be observed under light and phase contrast microscopy (video images can be supplied upon request).

SEM also revealed the presence of mucus and cilia on the apical cell surface of mature epithelial layers (Figure 6g, h, i, j). A relatively large population of ciliated cells was usually observed quite early in P_0 _ALI-cultures (even within the first week of ALI for those layers retaining morphological integrity post-switch), while in P_1 _cultures, ciliated cells appeared generally at around d 30 of ALI. The number of ciliated cells in P_1 _was lower than in P_0 _cultures but increased with age of the ALI-cultures (Figure 6i, j). Interestingly, many apical microvilli were detected by SEM in mature ALI-cultures, located either on ciliated or non-ciliated cells (Figure 6k, l), apparently larger in number and size than those observed in submerged cultures before ALI-creation. Moreover, in P_1 _ALI-cultures many areas of the surface in which microvilli appeared were elongated, showing, in some cases, a complex spatial arrangement (Figure 6l - inset). Finally, mature EBEC cultures immunostained for the cell-type specific CK 18 showed weak diffused positive signals; only some confined areas had high signal intensity (Figure 6m), while immunostaining for CK 10/13 was negative (Figure 6n).

## Discussion

In the present study, equine bronchial epithelial cells were isolated from bronchial branches of healthy horses and cultured on solid supports, under conventional submerged conditions or on semi-permeable transwell membrane inserts under ALI conditions. To the authors' knowledge, this is the first time that growth and differentiation of these cells were characterized extensively. This characterization comprised growth in the presence or absence of serum in defined AECGM, the comparison of primary and passaged cells, and the monitoring biochemical and functional expression of tight junctions. The limiting factor was to find out an optimal condition for EBECs with ultimate epithelial cell proliferation and differentiation properties.

Short-term (2 h) trypsin digestion of peeled and minced equine bronchial mucosa yielded large numbers of highly viable and pure epithelial cells. This isolation procedure and cell properties has been described recently for equine tracheal epithelial cells [21], and appears advantageous over isolation techniques used to isolate bronchial cells from intact bronchial segments of the horse [10,11] and other animal species [19,22,23]; weak enzymes and longer digestion time (16-48 h) were applied which presumably increase the number of other non-epithelial contaminating cells. Although trypsin is a strong dissociating enzyme, it did not affect the ability of EBECs to efficiently attach and replicate on collagen-coated plastic substratum. Freshly isolated EBECs reached confluence in less than 5 days, in agreement with previous reports for equine [10,11,24] and human [19,22,23,25] tracheobronchial epithelial cells.

The presence or absence of serum in AECGM did not affect the growth rate of primary EBECs on solid supports, but striking morphological and functional differences were observed. When grown in defined serum-free AECGM, EBECs exhibited an almost undifferentiated and migratory phenotype, similar to that reported for primary serum-free solid cultures of equine tracheal [24] and human bronchial [26] epithelial cells. Addition of 10% serum facilitated morphological and functional differentiation of EBECs as determined by the following phenotypes: 1) serum triggered more dome and cilia formation, cell-cell-contact and mucociliary differentiation that were retained over at least for 30 d [24,27]; similar to what is reported for primary outgrown explants of tracheal, bronchial and nasal epithelial cells [25,26,28]; 2) serum contributed to the formation of the cobblestone pattern and the continuous expression of ZO-1 and enhanced mucociliary EBEC differentiation; the latter confirmed by the apparent higher PAS-positivity in serum-grown EBEC cultures than those in serum-free. These findings suggest that serum constituents appeared to be necessary for the development of the complete differentiation potential as was described for airway epithelial cells in human and other species [3,16,29-33]. These factors may include retinoid acid or high calcium concentrations as described previously [22,34].

While the defined and serum-free AECGM was sufficient to culture both primary and passaged EBECs on solid supports, this same medium was not suitable for sustaining proliferation of these cells on membrane insert that allow differentiation of EBECs. In contrast to other studies, even seeding cells at high density in medium without serum was not sufficient to promote cell growth on porous membranes. The addition of 10% bovine calf serum to our defined supplemented AECGM was crucial and has led, even if latency was seen in P_1_, to rapid proliferation and differentiation of EBECs and sustaining the cultures even for over 90 d. Effective proliferation of equine bronchial [35] and human tracheo-bronchial epithelial cells [19,22] has been reported to occur at LLI in serum-free media. This contrast in observations may stem from differences in serum factors including calcium, albumin, and other adhesion molecules that are necessary for the proliferation, differentiation and long-term survival of EBECs [17,18,34,36]. Replacement of FBS with Ultroser G in a simple DMEM/Ham's F-12 medium plus retinoic acid also facilitated cell proliferation and differentiation when EBEC cultured at ALI [16,30,33].

Further, a striking observation in the present study was the difference in bioelectrical properties (TEER) obtained from P_0 _and P_1 _EBEC ALI cultures. First, in submerged culture conditions (LLI), TEER was much higher in P_0 _than in P_1 _EBEC cultures. This was consistent with the difference in growth rate observed in primary and passaged EBECs on solid supports. This difference may be due the selection of different epithelial cell populations during the passaging process. Subsequently, creation of ALI-culture conditions depended upon achievement of confluence for P_0 _cultures and upon achievement of a TEER threshold value of 50-100 Ω·cm^2 ^for P_1 _cultures, an indicator for the presence of functional tight junctions [37]. When switching from LLI to ALI, TEER values in P_1 _culture gradually increased and were higher and more stable than in P_0 _cultures. This observation suggests that passaged cells establish better cell-cell-connection than freshly isolated cells (P_0_) because P_0 _cells may be more heterogeneous and P_1 _cultures completely dedifferentiate to form tight monolayers as was observed for and ALI-cultures from other species [19,27].

TEER values from P_0 _or P_1 _EBECs were similar to those found in bronchial cells of other species and the immunostaining for ZO-1 was uniform in both P_0 _and P_1 _cultures. Therefore, it is not clear what affects the TEER value in the P_0 _and P_1 _cultures [38]. One can speculate that tight junction proteins other than ZO-1 must be involved in establishing tight cell-to-cell contact [20,39]. Moreover, the large inter-individual differences in TEER values measured among EBEC cultures established from different horses is an interesting feature. This suggests that polymorphic gene differences may exist in proteins that constitute the tight junction formation. Future studies should consider screening for such polymorphisms; given that the equine genome has now been sequenced and studies to identify such inter-individual differences are feasible.

Finally, in contrast to EBEC cultures on solid supports, P_0 _and P_1 _EBECs cultured on membrane inserts developed in a time-dependent manner surface structures, i.e. microvilli, microvilli-like structures and cilia, suggesting the importance of ALI conditions for establishing a state of differentiation that resembles the *in vivo *condition. In P_1 _EBEC ALI-cultures, cilia and microvilli were low in number and appeared in a delayed manner compared to P_0 _cells similar to what was observed in passaged airway epithelial cells in other species [15,27,40,41].

Cytokeratin staining for different epithelial cell population indicated the maturity of EBECs in ALI-culture. The broad spectrum antibody anti-human CK 5/6/18 stained positive P_0 _and P_1 _cells, suggesting the co-existence of columnar (CK 18-positive) and basal (CK 5/6-positive) cells. However, the pure anti-human CK 18 provided few signals in EBEC cultures and the supra-basal cell marker anti-human CK 10/13 showed no signal, suggesting a pseudo-stratified organization of the epithelial layers.

We also recognize several limitations to our study. We used primary EBECs from several strains of horses that could be the basis for the observed variations in TEER values. Future studies will focus on using EBECs from horses with the same strain and age to reduce variability. In addition, comparison of EBECs from healthy horses and those with reactive airway disease will clarify whether disease conditions may vary the the proliferative ability of cells in culture.

## Conclusions

In conclusion, findings of the present study show that EBECs can be isolated with high yield purity and viability and can be successfully cultured and sub-cultured up to passage 2 on solid supports. Serum supplemented AECGM enhanced the growth, differentiation potential, and longevity of EBEC cultures. On solid supports these EBEC cultures differentiated to a mucociliary epithelium in ALI conditions and were maintained over 90 days. TEER assessment resulted in a marked difference between P_0 _and P_1 _EBEC ALI cultures, whereby for the latter TEER values were higher and more stable, suggesting the suitability for *in vitro *studies of airway diseases. This equine airway cell model may facilitate investigations of the role of bronchial epithelia in the patho-physiology of airway diseases of the horse (RAO) and screening of pharmacologic treatments that could translate to designing more effective studies to treat human bronchial diseases.

## Methods

### Isolation of equine bronchial epithelial cells

Adult horses slaughtered at local abattoirs around Leipzig (Germany) of different breed, age (range: 1.5 - 26 years; median: 15 years), and sex (6 geldings and 6 mares) were used as donors of bronchial tissues. Horses had no sign of cardio-pulmonary disorders at clinical examination before slaughtering and were not under any drug treatment. After a thorough gross-anatomical inspection, fresh lungs were removed and transported on ice to the laboratory within 1 - 2 h.

After removing the trachea, main bronchi at the level of bifurcation as well as 1^st ^to 3^rd ^bronchial generations (with an outer Ø up to 1.5 cm) were blunt stripped of attached lung parenchyma and collected in ice-cold Hanks' balanced salt solution (HBSS; Ca^2+^/Mg^2+ ^free; PAA Laboratories GmbH, Pashing, Austria). Bronchial segments were then cut into pieces of about 5 cm in length, cut open lengthways and washed 2-3 times in ice-cold HBSS to remove blood, mucus and debris.

The isolation of primary bronchial epithelial cells was carried out under aseptic conditions as recently described for equine tracheal epithelial cells [21]. In brief, strips of bronchial mucosa were peeled off the sub-mucosa using sterile forceps and scalpel, collected in ice-cold HBSS, rinsed several times with the same buffer and minced manually into small pieces (about 1-2 mm^2^). Batches of about 500 mg minced tissue were transferred into 10 ml of digestion solution (0.25% trypsin - 0.6 mM EDTA solution in HBSS; Sigma-Aldrich, Deishenhofen, Germany) in sterile Erlenmeyer flasks and incubated at 37°C for 2 h in 5% CO_2 _atmosphere under gentle agitation (100 rpm). Enzymatic digestion was stopped by adding 5 ml of ice-cold 20% foetal bovine serum (FBS; Gibco-Invitrogen Life Technologies, Karlsruhe, Germany) in HBSS. After gently mixing, the crude cell-containing suspension was filtered through sterile double-layered gauze and rinsed twice with 5 ml of ice-cold HBSS. Cell suspension was then further sieved through sterile nylon strainers (pore size: 40 μm) (BD Biosciences, Franklin Lakes, NJ, USA) and rinsed twice with 5 ml of ice-cold HBSS. Dissociated cells were pelleted at 200 × *g*, 4°C for 10 min, and re-suspended in culture medium (see below). Cell number and viability was determined using Neubauer cell counting chamber and by trypan blue exclusion test. Before culturing or cytocentrifugation for immunocytochemical staining, cell density was adjusted in the indicated medium.

### Culture media

For culturing primary EBECs the following media were used:

1) Basal serum-free airway epithelial cell growth medium (AECGM) supplemented with bovine pituitary extract (0.4%), human recombinant epidermal growth factor (10 ng/ml), epinephrine (0.5 μg/ml), hydrocortisone (0.5 μg/ml), human recombinant insulin (5 μg/ml), triiodo-L-thyronine (6.7 ng/ml), transferrin (10 μg/ml) and retinoic acid (0.1 ng/ml). Both basal medium and supplements were from PromoCell GmbH, (Heidelberg, Germany).

2) Complete basal AECGM to which 10% FBS was added.

3) ALI-Medium, consisting of the nutrient mixture of DMEM/Ham's F-12 medium (1:1 vol/vol) with L-glutamine (4 mM) (PAA Laboratories GmbH, Pashing, Austria) supplemented with the serum substitute 2% Ultroser^®^-G (Pall BioSepra, Cergy-Saint-Christophe, France) and 15 ng/ml retinoic acid (Sigma-Aldrich, Deishenhofen, Germany).

All these media contained penicillin (200 U/ml), streptomycin (200 μg/ml) and amphotericin B (2.5 μg/ml) (PAA Laboratories GmbH, Pashing, Austria).

### EBEC culture on solid supports

Freshly dissociated EBECs were grown on tissue culture-treated plastic 75 and 25 cm^2 ^flasks, 94-mm Ø dishes or 24-well plates (Greiner Bio-One, Frickenhausen, Germany); moreover, for histological and immunocytochemical staining, cells were cultured also on glass cover slips in 24-well plates. Culture growth surfaces were coated with rat tail collagen type I (10 μg/cm^2 ^surface area; Sigma-Aldrich, Deishenhofen, Germany) and EBECs plated at a density of 5 × 10^5 ^viable cells/cm^2 ^in serum-free or serum-containing AECGM and maintained at 37°C in a humidified atmosphere of 5% CO_2_. The culture medium was changed initially after 24 h and then every 2-3 days. Cell morphology was regularly examined by a light/phase contrast inverted Olympus research microscope CKX41 (Olympus Optical Co. Ltd, Tokyo, Japan) and images were taken using a Canon DD60 U-PM TVC digital camera.

Primary (P_0_) EBEC cultures grown on tissue culture dishes or flasks in either media were sub-cultured when approximately 80 - 90% confluence was reached. In brief, medium was removed; cells were washed twice with PBS and detached using 0.05% trypsin/EDTA (PAA Laboratories GmbH, Pashing, Austria) for about 15 min at 37°C. Trypsin activity was terminated by adding 10% FBS in DMEM (containing penicillin/streptomycin and amphotericin B) and immediate centrifugation (200 × *g*, 4°C, and 10 min). Pelleted cells were re-suspended in serum-free or serum-containing AECGM, counted and proved for cell viability, and then seeded either on new collagen-coated flasks as described above or on semi-permeable membrane inserts at air-liquid-interface (ALI) culture establishment (see below).

### ALI-culture freshly isolated (P_0_) and passaged (P_1_) EBECs

Freshly isolated EBECs were seeded at a density of 0.9-1.5 × 10^6 ^viable cells/cm^2 ^onto collagen-coated (25 μg/cm^2 ^of rat tail collagen type I; Sigma-Aldrich, Deishenhofen, Germany) semi-permeable membrane inserts (PET, Greiner Bio-one, Frickenhausen, Germany; 0.4 μm pore size, 0.65 cm diameter, 0.336 cm^2 ^surface area in tissue culture 24-well plates. Both sides of the inserts were filled with either serum-free or serum-containing AECGM; 0.3 ml and 1 ml were added into the apical and the basolateral chamber, respectively. Cells were incubated at 37°C in a 5% CO_2 _humidified atmosphere; medium was changed in both chambers 24 h post-seeding and then every 2-3 days. Under this liquid-liquid interface (LLI) condition, microscopic examination was routinely carried out and transepithelial electrical resistance (TEER) was measured at each medium change (see below). Once the cells had achieved confluence, an air-liquid-interface (ALI) condition was created by removing the medium from the apical chambers and replacing the medium in the basolateral chambers with 1 ml of ALI-Medium.

ALI-cultures were also established from sub-confluent primary EBEC cultures pre-cultured on solid supports as described above, i.e. from P_0 _to P_1_. ALI culture conditions at P_1 _were as similar as P_0 _ALI, but two conditions were different: a) cultured P_0 _EBECs were seeded at higher density (1.5 - 2.2 × 10^6 ^viable cells/cm^2^); b) in P_1 _insert cultures an ALI was created, independently from confluence level, when, TEER reached a threshold value of 50-100 Ω·cm^2^.

### TEER measurements

TEER was measured in both P_0 _and P_1 _EBEC ALI-cultures as an index of tight junction and epithelial barrier function, using a volt-ohm-meter (Millicell^®^-ERS, Millipore Corp., Billerica, MA, USA).

Both the basolateral and apical chambers were filled with fresh pre-equilibrated medium and TEER was read after allowing the cell culture to get stable potential for about 15-30 min. Medium was removed from the apical side of the insert immediately after TEER readings to create again the ALI condition.

TEER values were corrected by subtracting the background resistance measured in only medium containing collagen-coated membrane inserts. These values were then converted to unit area resistance (Ω·cm^2^) by multiplying them with the effective membrane surface area (0.336 cm^2^). Data are expressed as mean ± SEM.

### Light microscopy and video imaging

Both solid and insert EBEC cultures were monitored routinely under bright field/phase contrast inverted microscope (Olympus CKX41; Olympus Optical Co. Ltd, Tokyo, Japan). Morphological changes and muco-ciliary differentiation were documented for each subject. Representative images were taken using a Canon DD60 U-PM TVC digital camera.

Moreover, video images were taken from EBECs grown on glass cover slips and membrane inserts to further prove ciliary beating and mucociliary differentiation. For this purpose, inverted Olympus CKX41 microscope was connected to CCD-camera (ORCA C4742-80 digital camera (Hamamutsa) and the images of active beating cilia were captured with AVI-Recorder and video clip was created.

### Scanning electron microscopy

SEM was performed to prove the occurrence of morphological markers of EBEC differentiation. From ALI cultures, membrane inserts were washed twice with PBS and fixed with 4% paraformaldehyde in PBS (Histofix; Carl Roth GmbH, Karlsruhe, Germany) at 4°C for 2 h or overnight. Insert samples were then rinsed 3 times × 10 min in 0.1 M PBS, post-fixed in 1% phosphate-buffered osmium tetroxide for 1 h at room temperature and rinsed again 3 times × 10 min in 0.1 M PBS. Then membranes were detached from the insert, dehydrated in graded ethanol solutions (starting with 30% up to absolute ethanol) and dried using a critical point dryer (CPD 030; BAL-TEC, Liechtenstein). To avoid rehydration, samples were kept in tubes filled with silica gel orange (Carl Roth GmbH, Karlsruhe, Germany) until sputter coating. Specimen were sputtered-coated with Gold-Palladium (90/10) at a specimen-target distance of 50 mm with approximately 40 mA for 60 sec (MED 020, BAL-TEC, Liechtenstein). SEM analysis was done with a LEO 1430 vp (Carl Zeiss, Oberkochen, Germany).

### Histology

To prove successful removal of epithelial cells, native and trypsin digested bronchial tissue samples and membrane inserts with EBECs cultures were washed in HBSS or PBS and fixed with 4% formaldehyde. All samples were then embedded in Paraplast, sectioned to a thickness of 3-4 μm and stained with Haematoxylin and Eosin (H&E).

### Periodic acid-Schiff staining

To demonstrate mucus cell differentiation, confluent layers of EBECs grown either on inserts at ALI or glass cover slips under conventional culture conditions were subjected to periodic acid-Schiff (PAS) staining to visualize neutral and acidic glycoproteins (likely mucins). Briefly, after discarding the culture medium and washing twice with PBS, samples were fixed with absolute ethanol for 10 min at room temperature, air-dried and finally stained as described in Romeis [42].

### Immunocytochemistry

Immunocytochemical fluorescence staining was carried out in: a) cytocentrifuged freshly isolated EBECs, b) EBECs cultured on either glass cover slips (at time of confluence) or c) on membrane inserts (at different time points of culture) for the following marker proteins:

- Cytokeratins 5/6/18 (CKs 5/6/18) label multiple epithelial cell types, all known to be expressed in cells of the human respiratory epithelium [43];

- Vimentin (VIM) labels mesenchymal cells such as fibroblasts;

- Cytokeratin 18 (CK 18), cell-type specific marker, is used as marker of columnar fully differentiated airway epithelial cells, either ciliated or secretory [44].

- Cytokeratins 10/13 (CKs 10/13), cell type-specific CK, as marker of suprabasal cells within normal stratified epithelia and shown to be expressed in human metaplastic respiratory epithelium [44].

- zonula occludens 1 (ZO-1, also known as tight junction protein 1 or TJP1), used as marker of intercellular tight junctions.

For immunocytochemical staining the following primary and secondary antibodies were used:

- *primary antibodies *- mouse monoclonal antibodies (mAbs) anti-human cytokeratins 5/6/18 (clone LP34; diluted 1:100 to use in preparation types a and b (see above), 1:10 to use in preparation type c, anti-human cytokeratins 10/13 (clone DE-K13; 1:10 diluted), anti-human cytokeratin 18 (clone DC 10; 1:10 diluted) and mouse anti-bovine vimentin (clone Vim 3B4; diluted 1:100 to use in preparation types a and b, 1:10 to use in preparation type c. All these antibodies were from Dako Deutschland GmbH (Hamburg, Germany); rabbit mono-specific antibody (msAb) anti-human tight junction protein 1 (TJP1; 1:50 diluted) from Sigma-Aldrich (Deishenhofen, Germany).

- *secondary antibodies *- goat anti-mouse IgG (Dako Deutschland GmbH, Hamburg, Germany; diluted 1:200 to use in preparation types a and b, 1:20 for use in preparation type c and goat anti-rabbit IgG (Sigma-Aldrich, Deishenhofen, Germany; 1:100 diluted), all fluorescein isothiocyanate (FITC)-labelled.

Cytocentrifuged fresh cells and confluent EBECs cultured on glass cover slips or membrane inserts were washed twice with PBS after medium removal, and fixed in ice-cold acetone or methanol for 5-10 min at -20°C (acetone fixation used for all CK and VIM detection, methanol for ZO-1 detection). Samples were washed in PBS, neutralized in 50 mM NH_4_Cl in PBS for 10 min at room temperature (RT), blocked in 3% bovine serum albumin (BSA) in PBS (blocking buffer) overnight at 4°C. Samples were then incubated for 1 h at RT with the primary antibody. After washing 3 times in PBS at 5 min intervals, specimens were incubated for 1 h at RT under light exclusion with secondary antibody. For nuclear (DNA) staining, 1 mg/ml stock solution of 4',6-diamidino-2-phenylindole-dihydrochloride (DAPI; Sigma-Aldrich, Deishenhofen, Germany) was added into diluted secondary Ab solution at final dilution of 1:2000 (type *a *and *b *cell preparations) or 1:600 (type *c *cell preparation). After further extensive washing in PBS (3 × 5 min), immunostained cell preparations were mounted with antifade mounting medium (Fluoromount-G, Southern Biotech, Birmingham, Alabama, USA) and observed under Olympus BX50 fluorescence microscope, equipped with a CC12 digital camera (Olympus Optical Co. Ltd, Tokyo, Japan). Blocking buffer was used to dilute primary and secondary antibodies. In all conditions, negative controls were prepared by omitting primary antibodies.

## Authors' contributions

GA, CZ, VA and FRU were involved in the concept and design of the experiments. GA, CZ, JK, CE, VA, JF, HAS, JS, YT and FRU were involved analysis, interpretation of data and helped to draft the manuscript. GA, CZ, YT, VA and FRU coordinated and prepared the manuscript. All authors read and approved the final manuscript.

## Authors' information

CZ present address: University of Bari, Faculty of Veterinary Medicine, Department of Veterinary Public Health, Division of Veterinary Pharmacology and Toxicology, Strada Prov.le per Casamassima, km 3, 70010 Valenzano (BA), Italy
